# Polypharmacy and Polymorbidity in Older Adults in Brazil: a public health challenge

**DOI:** 10.1590/S1518-8787.2016050006145

**Published:** 2016-12-01

**Authors:** Luiz Roberto Ramos, Noemia Urruth Leão Tavares, Andréa Dâmaso Bertoldi, Mareni Rocha Farias, Maria Auxiliadora Oliveira, Vera Lucia Luiza, Tatiane da Silva Dal Pizzol, Paulo Sérgio Dourado Arrais, Sotero Serrate Mengue

**Affiliations:** I Departamento de Medicina Preventiva. Escola Paulista de Medicina. Universidade Federal de São Paulo. São Paulo, SP, Brasil; II Departamento de Farmácia. Faculdade de Ciências da Saúde. Universidade de Brasília. Brasília, DF, Brasil; III Departamento de Medicina Social. Faculdade de Medicina. Universidade Federal de Pelotas. Pelotas, RS, Brasil; IV Departamento de Ciências Farmacêuticas. Centro de Ciências da Saúde. Universidade Federal de Santa Catarina. Florianópolis, SC, Brasil; V Departamento de Política de Medicamentos e Assistência Farmacêutica. Escola Nacional de Saúde Pública Sérgio Arouca. Fundação Oswaldo Cruz. Rio de Janeiro, RJ, Brasil; VI Departamento de Produção e Controle de Medicamentos. Faculdade de Farmácia. Universidade Federal do Rio Grande do Sul. Porto Alegre, RS, Brasil; VII Departamento de Farmácia. Faculdade de Farmácia, Odontologia e Enfermagem. Universidade Federal do Ceará. Fortaleza, CE, Brasil; VIII Programa de Pós-Graduação em Epidemiologia. Faculdade de Medicina. Universidade Federal do Rio Grande do Sul. Porto Alegre, RS, Brasil

**Keywords:** Aged, Comorbidity, Polypharmacy, Aging, Chronic Disease, Population Surveys

## Abstract

**OBJECTIVE:**

To analyze variations in the prevalence of chronic use of medicines by older adults in Brazil according to its possible association with the most prevalent chronic diseases and demographic and health factors, and to identify risk factors for polypharmacy.

**METHODS:**

A study based on data from the National Survey on Access, Use and Promotion of Rational Use of Medicines (PNAUM), a cross-sectional, population-based survey with probability sampling in Brazilian urban areas. The independent variable was the number of chronic-use medicines taken by older adults, linked to eight chronic diseases investigated. The intervening variables were gender, age group, marital status, level of education, socioeconomic status, Brazilian region, body mass index, smoking, self-perceived health, hospitalization in the previous year and having health insurance, besides the investigated chronic diseases. A multivariable analysis identified risk factors for polypharmacy.

**RESULTS:**

Prevalence of at least one chronic-use medicines among older adults was 93.0%. Of the total number of older adults, 18.0% used at least five medications (polypharmacy). Polypharmacy was higher among the oldest individuals (20.0%), in the South region (25.0%), in those with poor self-perceived health (35.0%), in obese individuals (26.0%), in those with reported health insurance (23.0%) or hospitalization in the previous year (31.0%), and among those who reported any of the investigated diseases, particularly diabetes (36.0%) and heart diseases (43.0%). The variables remaining in the final risk model for polypharmacy were age, region, perceived health, health insurance, hospitalization in the previous year and all investigated diseases except stroke.

**CONCLUSIONS:**

Older adults with specific diseases have risk factors for polypharmacy modifiable by actions aimed at the rational use of medicines. With the current population aging and successful drug access policy, the trend is an increase in drug use by older adults, which should feature as a priority in the planning agenda of the Brazilian Unified Health System (SUS).

## RESUMO

**OBJETIVO:**

Analisar as variações da prevalência do uso crônico de medicamentos por idosos no Brasil segundo sua possível associação com as doenças crônicas mais prevalentes, fatores sociodemográficos e de saúde, e identificar fatores de risco para polifarmácia.

**MÉTODOS:**

Estudo com dados da Pesquisa Nacional de Acesso, Utilização e Promoção do Uso Racional de Medicamentos, de caráter transversal e amostra probabilística populacional em municípios brasileiros urbanos. A variável independente foi o número de medicamentos de uso crônico por idosos, vinculados às oito doenças crônicas investigadas. As variáveis intervenientes foram sexo, faixa etária, situação conjugal, escolaridade, nível socioeconômico, região do País, índice de massa corporal, hábito de fumar, percepção da própria saúde, internação no último ano e posse de plano de saúde privado, além das doenças crônicas referidas. Uma análise multivariável identificou os fatores de risco para polifarmácia.

**RESULTADOS:**

A prevalência de pelo menos um medicamento de uso crônico entre idosos foi de 93,0%. Do total de idosos, 18,0% utilizavam pelo menos cinco medicamentos (polifarmácia). A polifarmácia foi maior entre os mais idosos (20,0%), na região Sul (25,0%), nos que avaliaram a própria saúde como ruim (35,0%), nos obesos (26,0%), nos que referiram ter plano de saúde (23,0%) ou internação no último ano (31,0%) e entre os que referiram qualquer uma das doenças investigadas, particularmente diabetes (36,0%) e doenças cardíacas (43,0%). No modelo final de risco para polifarmácia permaneceram idade, região, percepção de saúde, posse de plano de saúde, internação no último ano e todas as doenças investigadas exceto acidente vascular cerebral.

**CONCLUSÕES:**

Idosos com doenças específicas têm fatores de risco para polifarmácia modificáveis por ações que visem o uso racional de medicamentos. Com o envelhecimento populacional em curso e a política exitosa de acesso a medicamentos, a tendência é aumentar a utilização de medicamentos por idosos, que deve ser prioridade na agenda de planejamento do Sistema Único de Saúde.

## INTRODUCTION

The Brazilian population is aging fast. Brazil has currently about 16 million older adults, and by 2025 will have about 32 million, which will be the sixth largest population of older adults worldwide^23^ ^,^ ^24^. Population aging, combined with the epidemiological transition, increases the prevalence of non-communicable diseases (NCD), which, with the decline of communicable diseases as causes of death, have become the main causes of morbidity and mortality in Brazil^6^ ^,^ ^24^.

The *Política Nacional de Promoção da Saúde* (National Policy for Health Promotion), which began in 2006, proposes to control NCD by promoting health and prevention through programs that encourage a healthy lifestyle^20^. On the other hand, data from the 2013 *Pesquisa Nacional de Saúde* (National Health Survey) suggest that around 60 million Brazilians have at least one NCD and the majority resort to chronic-use medicines to control their diseases and thus not compromise their quality of life^19^ ^,^ ^a^.

The Brazilian Unified Health System (SUS), through pharmaceutical services, is responsible for guaranteeing access to medicines and promoting their rational use^18^. Regulated in 2011, the *Relação Nacional de Medicamentos Essenciais* (RENAME –National List of Essential Medicines) lists and standardizes medicines indicated to treat diseases or health conditions provided by the SUS network^b^. Older adults, who often suffer from polymorbidity^6^ ^,^ ^23^, usually take many medications, which may lead to inappropriate use of medicines and a higher incidence of side effects, avoidable by rational use of medicines^12^ ^,^ ^22^ ^,^ ^26^ ^,^ ^27^ ^,^ ^29^. Given the demographic imperative, a significant growth in the use of chronic medications by older adults is predicted, tending to increase as access improves.

Population studies on drug use in Brazil show that old age is indeed one of the main risk factors for being a heavy user of chronic-use medicines^1^ ^,^ ^3^ ^,^ ^7^ ^,^ ^13^ ^,^ ^21^. However, most population studies on drug use carried out in Brazil, which included older adults, investigated the use or not of a given medication either on the day of the interview^5^ ^,^ ^8^ ^,^ ^15^ ^,^ ^21^, or in the previous seven days^10^ ^,^ ^11^, or in the previous 15 days or more^1^ ^,^ ^3^ ^,^ ^4^ ^,^ ^14^ ^,^ ^25^ ^,^ ^28^. Only one of those studies specified the long-term use of medications^9^. None of the studies linked drug use to a chronic disease the respondent had been informed about by a doctor.

PNAUM is an important initiative of the Brazilian Ministry of Health in the field of pharmaceutical services, aimed at guiding the planning of pharmaceutical services for older adults and medical protocols for NCD control among older adults in Brazil. It sought to identify regional, sociodemographic and health particularities of the population associated with the chronic use of medicines by older adults.

This article aimed to describe the relationship of chronic use of medicines with the most prevalent NCD among older adults and with polymorbidity (the sum of those most prevalent diseases). It also seeks to understand the sociodemographic factors and health indicators associated with polypharmacy (use of at least five medicines), a sign of high use of medicines, which does not necessarily indicate irrational use, especially among older adults^5^ ^,^ ^22^ ^,^ ^27^.

## METHODS

PNAUM was a cross-sectional, population-based study carried out between September 2013 and February 2014. A national probability sample (n = 41,433), with a complex design, ensured representation in the five Brazilian regions, with stratification by gender and age. The strategy used was face-to-face interviews in households with data recorded in tablets with specific software for the survey’s questionnaires. Details of the methodology can be found in a specific publication^16^.

This study analyzed the information obtained from the population of older adults (aged 60 or over) (n = 9,019). Data on use of and access to chronic-use medicines were obtained from the report of previously informed diagnoses (“has any doctor said that you have…), possible indication for treatment (“has any doctor indicated treatment for...”), and the naming of medicines used for each disease (compared with prescriptions and packaging, whenever possible). Eight chronic diseases were selected to be investigated, namely: high blood pressure, diabetes mellitus, heart diseases, high blood cholesterol, medical history of stroke, chronic lung diseases, rheumatism and depression. Other chronic diseases occasionally reported were not included in the analysis because they represented a very large and diverse group of diseases, with very low frequencies. The variable chronic polymorbidity was created for purposes of analysis, ranging from zero to eight, related to the above mentioned diseases.

The dependent variable was the number of chronic-use medicines used by older adults, linked to one of those eight chronic diseases reported. Only specific medicines for each one of the diseases were considered^c^. There is no universally accepted definition for polypharmacy. The option in this study was to work with five or more medicines associated with one of the eight chronic diseases, in the knowledge that other studies consider polypharmacy as taking more than three or sometimes more than 10 medicines^26^ ^,^ ^27^.

As intervening variables, the study analyzed the sociodemographic characteristics of respondents – gender, age group, marital status, level of education, socioeconomic status according to the *Critério Classificação Econômica Brasil* (CCEB/ABEP, 2013 – Brazilian Economic Classification Criterion) in brackets A/B; C; D/E (http://www.abep.org/criterio-brasil), and Brazilian region (North, Northeast, South, Southeast and Midwest) – and a few health indicators – body mass index (BMI with specific cutoffs for older adults), current smoking habits, self-perceived health, hospitalization in the previous year and having health insurance, in addition to the above mentioned diseases. Pearson’s Chi-squared test was used to verify associations. Poisson regression, univariate and multivariable, was performed to identify the most significant factors associated with a high prevalence of polypharmacy (≥ 5 chronic medications). The variables with p-value lower than 0.20 in the univariate analyses were selected for the multivariate analysis. Variables with p-value lower than 0.05 remained in the model. The analyses were performed using the SPS v. 18 statistical package and the results were weighted according to the sample design. The study was approved by the *Conselho Nacional de Ética em Pesquisa* (National Research Ethics Council – Opinion 398.131, from September 16, 2013).

## RESULTS

Table 1 features the age composition of the sample according to the sociodemographic and health variables evaluated. Just over half of the older adults were under 70 years old, about one third were between 70 and 79 years, and 14% were 80 or older. More than half were women, with no significant differences between age groups. Most lived with a spouse, a condition significantly more prevalent in the group aged 60-69 (64%) and less so in the group aged 80 years or over. In general, the level of education was low. Most of the older adults were in economic bracket C (55%), while about 24% were in brackets D and E. Most of the older adults lived in the Southeast region (53%), followed by the Northeast (21%). A minority of older adults reported smoking (11%), which tended to be lower in the group aged 80 or over (6%). About 25% of the older adults were overweight or obese, but the group aged 80 or over showed a trend towards low weight (30%). Only 10% of the older adults reported hospitalization in the previous year, with a significant increase in prevalence in the ≥ 80 group (15%). In 27% of cases the older adults had health insurance.

**Table 1:** Sociodemographic and health profile of older adults by age groupa. PNAUM, Brazil, 2014. (N = 6,844)

Variable	Age group (years)			Total	p

	60–69	70–79	≥ 80		
		
	% (95%CI)	% (95%CI)	% (95%CI)		
Gender					0.094
Male	43.7 (41.8–45.6)	40.6 (38.4–42.9)	40.6 (36.5–44.9)	42.2 (40.7–43.8)	
Female	56.3 (54.4–58.2)	59.4 (57.1–61.6)	59.4 (55.1–63.5)	57.8 (56.2–59.3)	
Marital status					< 0.001
Partner	64.0 (61.8–66.1)	52.6 (49.8–55.4)	34.8 (30.4–39.5)	55.8 (53.9–57.7)	
No partner	36.0 (33.9–38.2)	47.4 (44.6–50.2)	65.2 (60.5–69.6)	44.2 (42.3–46.1)	
ABEP^b^					0.005
A/B	23.1 (20.7–25.7)	19.3 (16.7–22.2)	21.7 (17.9–26.1)	21.7 (18.5–24.0)	
C	55.2 (52.9–57.5)	55.4 (52.2–58.5)	51.3 (47.1–55.5)	54.7 (52.7–56.6)	
D/E	21.7 (19.8–23.7)	25.3 (22.5–28.4)	27.0 (23.4–30.8)	23.7 (21.8–25.7)	
Level of educations (in years)					0.003
0	13.9 (12.4–15.6)	15.2 (13.2–17.4)	20.8 (17.5–24.5)	15.4 (13.9–16.9)	
1-8	44.7 (42.3–47.1)	42.9 (40.3–45.5)	41.6 (36.6–46.7)	43.6 (41.8–45.5)	
≥ 8	41.4 (38.9–44.0)	42.0 (39.2–44.8)	37.7 (33.4–42.1)	41.0 (39.2–42.9)	
Brazilian region					0.034
North	5.2 (4.1–6.6)	4.6 (3.5–6.0)	4.1 (3.1–5.5)	4.8 (3.8–6.1)	
Northeast	20.6 (16.7–25.1)	21.6 (17.3–26.6)	22.7 (17.9–28.4)	21.2 (17.2–25.8)	
Southeast	51.4 (45.4–57.4)	53.1 (46.7–59.5)	54.9 (47.7–61.9)	52.5 (46.5–58.4)	
South	15.5 (12.5–19.1)	14.3 (11.2–18.0)	12.7 (9.6–16.6)	14.7 (11.8–18.1)	
Midwest	7.3 (5.8–9.3)	6.4 (5.0–8.3)	5.6 (4.2–7.5)	6.8 (5.4–8.5)	
BMI					0.247
Low	19.9 (18.3–21.5)	22.4 (20.3–24.7)	29.6 (26.5–33.0)	22.4 (21.1–23.6)	
Normal	53.4 (51.2–55.6)	54.0 (51.5–56.4)	49.2 (44.7–53.8)	52.9 (51.4–54.4)	
Overweight	10.6 (9.5–11.9)	10.3 (8.8–11.9)	10.3 (6.9–15.0)	10.5 (9.5–11.5)	
Obese	16.1 (14.5–17.7)	13.3 (11.5–15.3)	10.9 (8.5–13.8)	14.3 (13.1–15.5)	
Current smoker					< 0.001
Yes	12.4 (11.1–13.9)	9.4 (8.0–11.1)	5.7 (4.1–7.8)	10.6 (9.6–11.6)	
No	87.6 (86.1–88.9)	90.6 (88.9–92.0)	94.3 (92.2–95.9)	89.4 (88.4–90.4)	
Self-perceived health					< 0.001
Very good or good	60.3 (57.9–62.7)	55.2 (52.6–57.7)	49.0 (45.3–52.7)	56.9 (55.0–58.8)	
Average	33.7 (31.5–35.9)	37.6 (35.1–40.1)	40.4 (37.2–43.7)	36.0 (34.4–37.6)	
Poor or very poor	6.0 (5.1–7.1)	7.3 (5.9–8.9)	10.6 (8.6–13.0)	7.1 (6.4–7.9)	
Health insurance					< 0.002
Yes	24.8 (22.0–27.9)	29.0 (25.7–32.5)	30.6 (26.3–35.4)	27.0 (24.3–29.9)	
No	75.2 (72.1–78.0)	71.0 (67.5–74.3)	69.4 (64.6–73.7)	73.0 (70.1–75.7)	
Hospitalization in previous 12 months					< 0.001
Yes	8.5 (7.4–9.7)	10.3 (8.7–12.2)	14.6 (12.1–17.6)	10.0 (9.1–11.0)	
No	91.5 (90.3–92.6)	89.7 (87.8–91.3)	85.4 (82.4–87.9)	90.0 (89.0–90.9)	
High blood pressure					< 0.001
Yes	47.2 (45.2–49.3)	34.6 (32.1–37.0)	33.6 (30.0–37.3)	41.0 (39.5–42.6)	
No	52.8 (50.7–54.8)	65.4 (63.0–67.9)	66.4 (62.7–70.0)	59.0 (57.4–60.5)	
Diabetes mellitus					0.018
Yes	81.8 (80.1–83.1)	78.5 (76.5–80.5)	82.1 (79.0–84.8)	80.8 (79.6–82.0)	
No	18.2 (16.8–19.6)	21.5 (19.5–23.5)	17.9 (15.2–21.0)	19.2 (18.0–20.4)	
Heart disease					< 0.001
Yes	88.8 (87.6–89.9)	82.2 (79.7–84.3)	78.3 (74.9–81.3)	85.0 (84.0–86.1)	
No	11.2 (10.1–12.4)	17.8 (15.7–20.3)	21.7 (18.7–25.1)	15.0 (13.9–16.0)	
High blood cholesterol					0.129
Yes	77.7 (75.9–79.3)	75.2 (72.5–77.8)	78.6 (75.1–81.7)	77.0 (75.4–78.5)	
No	22.3 (20.7–24.1)	24.8 (22.2–27.5)	21.4 (18.3–24.9)	23.0 (21.5–24.6)	
Stroke					< 0.001
Yes	95.5 (94.6–96.2)	94.9 (93.7–95.9)	90.0 (87.9–91.8)	94.5 (93.9–95.0)	
No	4.5 (3.8–5.4)	5.1 (4.1–6.3)	10.0 (8.2–12.1)	5.5 (5.0–6.1)	
Lung disease					0.071
Yes	96.0 (95.2–96.7)	94.6 (93.3–95.6)	94.3 (91.7–96.1)	95.3 (94.6–95.9)	
No	4.0 (3.3–4.8)	5.4 (4.4–6.7)	5.7 (3.9–8.3)	4.7 (4.1–5.4)	
Rheumatism					< 0.001
Yes	85.5 (83.7–87.2)	80.4 (78.1–82.6)	82.8 (79.7–85.5)	83.5 (82.0–84.8)	
No	14.5 (12.8–16.3)	19.6 (17.4–21.9)	17.2 (14.5–20.3)	16.5 (15.2–18.0)	
Depression					0.676
Yes	90.6 (89.3–91.8)	89.9 (88.0–91.6)	91.1 (88.5–93.1)	90.5 (89.4–91.4)	
No	9.4 (8.2–10.7)	10.1 (8.4–12.0)	8.9 (6.9–11.5)	9.5 (8.6–10.6)	

ABEP: *Associação Brasileira de Empresas de Pesquisa* (Brazilian Association of Survey Companies); BMI: Body Mass Index

^a^ The percentages and confidence intervals were calculated based on the expanded sample – adjusted by sampling weights and post-stratification by age and gender.

^b^ 2013 *Critério Classificação Econômica Brasil* (Brazilian Economic Classification Criterion) of ABEP.

Prevalence of high blood pressure was 59% and increased significantly with age (66% in the ≥ 80 group), as did heart disease prevalence, which ranged from 11% in the group aged 60 to 69 to 22% in the group aged ≥ 80, and prevalence of stroke was reported by a minority (6%), reaching 10% in the group aged ≥ 80. Prevalence of the other diseases did not vary significantly with age.

The number of reported diseases per older adult ranged from zero to eight diseases; 26% did not report any of the eight diseases, 31% reported only one, 22%, two, 13%, three and 9% reported at least four. Among those that reported only one chronic disease (31%), in 66% of cases the disease was high blood pressure, 8%, diabetes, 5%, heart diseases, 7%, high blood cholesterol, 1%, stroke, 3%, lung diseases, 6%, rheumatism and 4%, depression (data not shown).

Of the total number of older adults, one-third, 31%, did not report any chronic medication for those diseases, ranging from 37% in the group aged 60 to 69 to 24% in the group aged 80 or over. Overall, 17% of older adults reported using one chronic drug, 17%, two, 21%, three or four, and 14% used at least five medicines (data not shown).

Table 2 analyzes the prevalence of chronic-use medicines for the eight chronic diseases, among the older adults who reported at least one of those diseases (74%), according to sociodemographic and health variables. Overall prevalence of at least one specific medicine for one of those diseases was 93%, with 23% using a single drug, 23% using two, 29% using three or four medications, and 18% using at least five and characterizing polypharmacy. Prevalence of polypharmacy was significantly higher among: female older adults (20%), those aged 70 to 79 (22%), resident in the South region (25%) (with prevalence of polypharmacy in the North region being noticeably low [3%]), those who reported having health insurance (22%), those who reported hospitalization in the previous year (32%), overweight individuals (25%), those with poor or very poor self-perceived health (37%), and those who reported each one of the eight diseases. Prevalence of polypharmacy varied from 21% in high blood pressure to 42% in heart diseases.

**Table 2:** Prevalence of older adults that utilize chronic-use medicines (CM) for at least one of the eight chronic diseases, according to sociodemographic and health variables. PNAUM, Brazil, 2014a.

Independent variable	Number of chronic–use medicines				

	zero	1	2	3 a 4	≥ 5
				
	% (95%CI)	% (95%CI)	% (95%CI)	% (95%CI)	% (95%CI)
Gender					
Male	9.0 (7.2–11.3)	25.0 (22.8–27.3)	25.8 (23.5–28.3)	24.6 (22.4–27.0)	15.6 (13.8–17.5)
Female	5.2 (4.3–6.3)	21.6 (19.5–23.8)	21.6 (19.8–23.5)	31.5 (29.3–33.8)	20.1 (18.4–21.9)
Age (years)					
60-69	8.0 (6.7–9.5)	25.0 (22.7–27.4)	24.1 (22.0–26.5)	27.1 (25.2–29.1)	15.8 (14.0–17.7)
70-79	5.3 (3.8–7.3)	20.9 (18.8–23.1)	21.9 (19.6–24.4)	30.4 (27.7–33.2)	21.5 (19.2–24.0)
≥ 80	5.7 (3.5–9.2)	20.9 (18.0–24.1)	23.0 (19.7–26.7)	30.9 (27.0–35.2)	19.5 (16.3–23.1)
Marital status					
Partner	7.1 (5.9–8.6)	23.6 (21.9–25.5)	24.1 (22.2–26.0)	27.5 (25.6–29.4)	17.7 (16.0–19.5)
No partner	6.2 (4.9–7.7)	22.0 (19.9–24.2)	22.1 (19.9–24.4)	30.6 (28.1–33.3)	19.2 (17.3–21.3)
ABEP^b^					
A/B	6.3 (4.6–8.6)	23.9 (21.0–27.1)	20.7 (18.2–23.4)	28.7 (25.5–32.3)	20.4 (17.5–23.6)
C	6.4 (5.3–7.7)	22.5 (20.5–24.5)	23.3 (21.3–25.4)	29.3 (27.0–31.7)	18.5 (16.8–20.3)
D/E	7.7 (5.5–10.7)	23.2 (20.5–26.0)	25.3 (22.3–28.5)	27.7 (24.9–30.7)	16.2 (13.7–19.0)
Level of educations (in years)					
0	8.1 (6.1–10.9)	22.1 (18.9–25.7)	23.0 (20.1–26.3)	28.8 (25.2–32.7)	18.0 (14.6–21.9)
1-8	6.6 (5.3–8.1)	23.8 (21.7–26.1)	23.9 (21.5–26.4)	28.0 (25.6–30.5)	17.7 (15.7–19.9)
≥ 8	6.2 (4.9–7.8)	22.4 (20.1–24.9)	22.5 (20.5–24.7)	29.7 (27.2–32.3)	19.2 (17.2–21.3)
Brazilian region					
North	10.3 (8.2–12.9)	46.2 (41.7–50.8)	22.6 (20.2–25.3)	17.6 (15.1–20.5)	3.2 (2.3–4.5)
Northeast	10.2 (8.5–12.3)	24.2 (22.2–26.4)	25.7 (23.4–28.2)	24.9 (22.2–27.7)	14.9 (12.8–17.3)
Southeast	5.7 (4.2–7.6)	21.2 (18.7–23.9)	23.0 (20.5–25.8)	31.1 (28.3–34.0)	19.0 (17.0–21.3)
South	4.4 (3.2–6.1)	20.4 (17.7–23.3)	20.8 (18.4–23.4)	29.4 (26.7–32.2)	25.1 (21.8–28.7)
Midwest	6.3 (4.8–8.3)	23.0 (20.3–26.0)	22.2 (19.3–25.5)	29.7 (26.8–32.8)	18.7 (15.8–22.0)
BMI					
Low	7.5 (5.3–10.4)	27.5 (24.5–30.7)	22.3 (19.5–25.4)	28.3 (25.2–31.6)	14.5 (11.9–17.6)
Normal	7.2 (5.7–9.1)	22.5 (20.6–24.6)	24.3 (22.3–26.5)	28.1 (26.1–30.2)	17.8 (16.1–19.8)
Overweight	4.9 (3.3–7.4)	23.1 (18.5–28.6)	23.8 (20.0–28.0)	29.5 (25.2–34.3)	18.7 (14.9–23.1)
Obese	3.5 (2.2–5.5)	16.1 (13.1–19.6)	22.4 (19.2–26.1)	32.5 (28.4–36.9)	25.4 (21.9–29.3)
Current smoker					
Yes	9.5 (6.9–12.9)	27.4 (23.0–32.3)	21.2 (17.4–25.7)	29.2 (24.4–34.6)	12.7 (9.5–16.7)
No	6.5 (5.5–7.7)	22.7 (21.0–24.5)	23.6 (21.9–25.4)	28.7 (26.8–30.6)	18.5 (17.1–20.1)
Self-perceived health					
Very good or good	8.2 (6.6–10.1)	28.5 (26.5–30.6)	25.4 (23.3–27.6)	26.3 (24.2–28.6)	11.6 (10.1–13.3)
Average	5.2 (4.2–6.4)	18.9 (16.6–21.4)	22.0 (20.0–24.1)	31.6 (29.2–34.0)	22.4 (20.3–24.7)
Poor or very poor	5.2 (3.6–7.5)	11.2 (8.4–14.7)	16.6 (13.4–20.4)	30.2 (25.7–35.0)	36.8 (31.5–42.5)
Health insurance					
Yes	5.1 (3.6–7.4)	21.3 (18.9–23.8)	21.4 (18.7–24.4)	30.0 (26.8–33.4)	22.2 (22.0–25.1)
No	7.3 (6.2–8.6)	23.6 (21.8–25.4)	23.9 (22.2–25.7)	28.4 (26.6–30.3)	16.8 (15.3–18.4)
Hospitalization					
Yes	4.0 (2.7–5.8)	13.5 (10.6–17.1)	20.0 (16.5–24.0)	30.2 (26.1–34.7)	32.3 (27.7–37.3)
No	7.1 (6.0–8.3)	24.2 (22.5–26.0)	23.6 (22.0–25.3)	28.8 (27.1–30.6)	16.3 (15.0–17.8)
High blood pressure					
Yes	21.1 (17.6–25.0)	29.2 (26.1–32.5)	22.2 (19.2–25.6)	19.0 (16.1–22.3)	8.5 (6.7–10.9)
No	2.9 (2.4–3.6)	21.3 (19.7–22.9)	23.4 (21.7–25.2)	31.4 (29.6–33.4)	20.9 (19.3–22.6)
Diabetes mellitus					
Yes	7.8 (6.6–9.0)	28.0 (26.2–29.9)	25.0 (23.3–26.9)	26.8 (24.9–28.9)	12.4 (11.1–13.7)
No	3.5 (2.4–5.2)	8.2 (6.7–10.0)	17.8 (15.3–20.7)	34.8 (31.8–38.0)	35.6 (32.7–38.6)
Heart disease					
Yes	7.5 (6.4–8.7)	26.7 (24.9–28.6)	25.4 (23.6–27.3)	28.1 (26.2–30.0)	12.3 (11.0–13.7)
No	3.4 (2.1–5.4)	7.8 (6.0–9.9)	14.2 (11.9–16.9)	32.1 (29.0–35.5)	42.4 (38.6–46.3)
High blood cholesterol					
Yes	7.3 (6.2–8.7)	28.3 (26.6–30.2)	26.4 (24.5–28.4)	25.5 (23.7–27.4)	12.4 (11.1–13.8)
No	5.2 (3.9–6.9)	10.8 (9.0–12.9)	16.0 (13.7–18.6)	36.3 (33.5–39.3)	31.7 (28.8–34.7)
Stroke					
Yes	6.7 (5.7–7.9)	23.9 (22.3–25.5)	23.8 (22.2–25.4)	28.5 (26.7–30.3)	17.2 (15.9–18.6)
No	6.0 (4.0–8.9)	10.7 (7.6–14.8)	16.0 (12.4–20.4)	33.7 (28.4–39.5)	33.6 (27.9–39.9)
Lung disease					
Yes	6.5 (5.6–7.7)	23.6 (22.0–25.3)	23.6 (22.1–25.3)	28.9 (27.1–30.6)	17.4 (16.0–18.8)
No	8.4 (3.8–17.6)	12.4 (8.9–17.0)	16.4 (12.4–21.3)	29.4 (23.7–35.9)	33.4 (26.6–41.0)
Rheumatism					
Yes	6.3 (5.2–7.6)	25.9 (24.0–27.8)	25.1 (23.3–27.0)	27.8 (26.0–29.7)	14.9 (13.6–16.4)
No	8.0 (6.3–10.0)	12.5 (10.3–15.3)	16.5 (14.2–19.0)	32.7 (29.8–35.7)	30.4 (27.2–33.7)
Depression					
Yes	7.1 (6.1–8.3)	24.9 (23.1–26.7)	24.4 (22.8–26.0)	27.9 (26.1–29.7)	15.8 (14.4–17.3)
No	3.5 (2.0–6.2)	9.5 (7.3–12.4)	15.1 (11.8–19.1)	35.9 (31.6–40.4)	36.0 (31.0–41.2)

Total	6.7 (5.7–7.8)	22.9 (21.4–24.5)	23.2 (21.6–24.8)	28.9 (27.2–30.6)	18.4 (17.0–19.8)

ABEP: *Associação Brasileira de Empresas de Pesquisa* (Brazilian Association of Survey Companies); BMI: Body Mass Index

^a^ The percentages and confidence intervals were calculated based on the expanded sample – adjusted by sampling weights and post-stratification by age and gender.

^b^ 2013 *Critério Classificação Econômica Brasil* (Brazilian Economic Classification Criterion) of ABEP.

Analyzing drug use in relation to polymorbidity, prevalence of polypharmacy was 3% among those who reported only one of the diseases, 13% among older people with two diseases, 37% among those with three diseases, and 60% among those who reported at least four diseases (data not shown).

Table 3 shows the outcome of the multivariate analysis controlling the use of five or more medications (polypharmacy) for those eight chronic diseases by all sociodemographic and health variables presented. The prevalence ratio for polypharmacy was marginally higher among the oldest individuals and those who reported hospitalization in the previous year (PR = 1.3), and significantly higher among those who perceived their own health as poor or very poor (PR = 1.7). Use was significantly lower in the North region, causing the prevalence ratio for polypharmacy in all other regions to range from 3.6 in the Northeast region to 5.1 in the South. All diseases except stroke remained in the final risk model for polypharmacy, with prevalence ratios ranging from 1.5 in rheumatism to 2.3 in diabetes and heart diseases.

**Table 3:** Multivariate model for risk factors for polypharmacy among older adults with at least one of the eight chronic diseases.

Variable	Category	PR	95%CI	p
Age	60-69	1.00		0.033
	70-79	1.20	1.04–1.38	
	≥ 80	1.08	0.90–1.30	
Region	North	1.00		< 0.001
	Northeast	3.61	2.56–5.09	
	Southeast	4.22	3.02–5.89	
	South	5.03	3.58–7.07	
	Midwest	3.89	2.74–5.51	
Perception	Very good or good	1.00		< 0.001
	Average	1.40	1.21–1.61	
	Poor or very poor	1.65	1.37–2.00	
Health Insurance	Yes	1.11	0.99–1.26	0.083
	No			
Hospitalization	Yes	1.28	1.10–1.49	0.001
	No			
High blood pressure	Yes	2.08	1.66–2.61	< 0.001
Diabetes mellitus	Yes	2.30	2.02–2.62	< 0.001
Heart disease	Yes	2.26	1.97–2.60	< 0.001
High blood cholesterol	Yes	1.76	1.55–2.01	< 0.001
Lung disease	Yes	1.29	1.03–1.61	0.024
Rheumatism	Yes	1.53	1.32–1.76	< 0.001
Depression	Yes	1.78	1.53–2.07	< 0.001

Among older adults who reported at least one of the eight chronic diseases, 583 specific medicines were reported to treat those diseases, which, in different dosage forms (including fixed-dose combinations), totaled 17,634 reports. The 40 most frequently cited medicines accounted for 73% of reports. In 63% of cases these were medicines for high blood pressure or heart diseases and cholesterol control; 13% for diabetes; and 13% were psychoactive. Table 4 features the 10 most reported medicines by older adults, accounting for 49% of reports. The single most reported drug, considering only the number of reports within each disease separately, and not including fixed-dose combinations, was hydrochlorothiazide (9%), followed by losartan (8%), both reported for high blood pressure, heart diseases or stroke. Simvastatin, indicated for blood cholesterol control, was the third most reported drug (6%), followed by metformin for diabetes control (5%). The list was completed with enalapril (4%), captopril (9%), atenolol (6%), glibenclamide (4%), propranolol (2%), and furosemide (2, 0%), all reported for treating high blood pressure, with the exception of glibenclamide, used to treat diabetes.

**Table 4:** Most commonly reported medicines (10) by older adults to specifically treat one of the eight chronic diseases mentioned.

Medicines	% of reports
Hydrochlorothiazide	8.3
Losartan	7.6
Simvastatin	6.7
Metformin	5.5
Enalapril	4.7
Captopril	4.5
Atenolol	3.7
Amlodipine	2.9
Acetylsalicylic acid	2.9
Glibenclamide	2.4

## DISCUSSION

Based on the literature review^4^ ^,^ ^5^ ^,^ ^9^ ^,^ ^10^ ^,^ ^13^ ^,^ ^21^ ^,^ ^25^ ^,^ ^28^, this is the first population study representative of the five Brazilian regions to assess the prevalence of chronic-use medicines among older adults (aged 60 or over), linking the medicines to a previous diagnosis of the most prevalent NCD among older adults (high blood pressure, diabetes, heart diseases, dyslipidemia, stroke, lung disease, rheumatism and depression).

The findings of this study showed that the vast majority of older adults (74%) reported at least one of the eight NCD mentioned, data compatible with population studies of older adults^14^ ^,^ ^23^. Of those older adults with chronic disease, the absolute majority (93%) used at least one chronic-use medicines. This prevalence of use of chronic-use medicines among older adults was higher than the prevalence reported in other nationwide (73% to 83%)^23^ ^,^ ^b^ or municipal (72% to 89%)^5^ ^,^ ^7^ ^,^ ^10^ ^,^ ^14^ ^,^ ^25^ studies, which did not specify chronic-use medicines and computed all reported medicines. This apparent paradox could be explained by the actual link between drug use and the existence of a chronic disease, which placed in the denominator only those who reported one of the selected NCD. Moreover, one can assume that the reports linked to the diseases may have prompted the memory of older adults concerning their list of chronic-use medicines.

On the other hand, prevalence of polypharmacy in the treatment of older adults with at least one of the eight diseases was 18%. This prevalence was much lower than that reported in other studies, which showed more than 35% of polypharmacy among older adults^5^ ^,^ ^27^. It should be noted that this study computed chronic-use medicines only, considered specific for each one of the eight diseases, which certainly underestimated the total number, since chronic-use medicines reported by respondents related to other diseases and those of occasional use were not included. Other studies, in turn, investigated the use of any medication by older adults, and not necessarily those of chronic use, for any disease, which tends to increase the numerator and thus the estimate of polypharmacy.

Women were the majority in the sample and had a higher prevalence of use and polypharmacy, as in most population studies with older adults^5^ ^,^ ^14^; however, the gender variable was not maintained as an independent factor of chronic use among older adults in the multivariate model discussed below. Prevalence of use of a chronic-use medicines significantly increased with age, reaching 95% in the group aged 80 or over. Polypharmacy was significantly higher in the group aged 70 to 79, but did not significantly increase in the group aged 80 or older, suggesting a selection of healthier older adults in higher age percentiles. This increased use among older people is also a common finding in almost all studies on drug use, especially when compared to youngsters and young adults^1^ ^,^ ^3^ ^,^ ^11^.

Most older adults lived with a spouse and showed a prevalence of use very similar to the average. Most studies with older adults show that living with a spouse is a condition that protects against the worsening of illnesses^5^ ^,^ ^23^. In the analysis by Brazilian regions, the North and Northeast regions showed lower use, in contrast to the South and Southeast regions, a fact that probably correlates with regional socioeconomic differences^4^ ^,^ ^5^ ^,^ ^14^ ^,^ ^28^. Nevertheless, the extremely low prevalence of polypharmacy in the North region (3%) stood out.

Having health insurance seemed to favor the use of medicines and polypharmacy, a finding that is supported by other studies^4^ ^,^ ^5^ ^,^ ^14^ ^,^ ^28^. Self-perception proved to be a good health indicator, as is common in studies with older adults^4^ ^,^ ^5^ ^,^ ^14^ ^,^ ^28^, with high prevalence of polypharmacy among older adults who rated their health as poor or very poor. Report of previous hospitalization identifies older adults with high prevalence of polypharmacy, a factor that was related to the prevalence of diseases and appears in other studies as a risk factor^4^ ^,^ ^5^ ^,^ ^14^ ^,^ ^28^.

In the multivariate analysis, controlling by all other intervening variables, all diseases, with the exception of stroke, significantly increase the risk of polypharmacy. In ascending order: lung disease (PR = 1.3), rheumatism (PR = 1.5), depression and high blood cholesterol (PR = 1.8), high blood pressure (PR = 2.1), and, heading the list, diabetes and heart diseases (PR = 2.3). It is evident that the presence of these prevalent chronic diseases among older adults is the greatest factor of drug use, serving as an indicator of risk groups easily identifiable by the health system. Polimorbidity summarizes the importance of these diseases among older adults, with prevalence of polypharmacy above 60% in the group with polymorbidity.

Obesity appeared as a factor of increased drug use, with 26% of polypharmacy among obese individuals, but this finding lost significance when controlled by the other chronic diseases. No studies were found suggesting the independence of this factor.

Only one study with older adults in São Paulo correlated drug use by older adults with pre-existent NCD^5^. The risk of polypharmacy significantly increased in the presence of diabetes, heart problems, high blood pressure, rheumatic disease and lung disease, in descending order of risk.

The variable region is noteworthy, appearing in all analyses as strongly associated with the use of chronic-use medicines to treat the investigated diseases and expressing regional differences that are independent of all other sociodemographic or health variables. The fact that the North region had a very low baseline value of polypharmacy (3%) must have weighed in the multivariate analysis, perhaps increasing the significance of differences, which was less pronounced between the other four regions. But it certainly makes one think of regionalized policies for rational use, improved access and increased adherence to treatment^29^.

It is remarkable that this high use of medicines by older adults can be summarized in a list of 32 single medicines that account for 77% of reports of use of chronic-use medicines by older adults. Prominent among them are medicines used to control high blood pressure (62%). Four medicines, two for high blood pressure (hydrochlorothiazide and losartan), one for high blood cholesterol (simvastatin) and one for diabetes (metformin), account for 48% of reports and 10 medicines account for 80% of reports in this group. It should be emphasized that all of the 10 most used medicines are listed in RENAME^b^ and can therefore be obtained via public pharmaceutical policies at no cost to the patient. All are part of the *Componente Básico da Assistência Farmacêutica* (Basic Pharmaceutical Services Component), can be obtained at SUS health units and are part of the *Programa Farmácia Popular* (Popular Pharmacy Program). Public access to these medicines is assured with a medical prescription, and is therefore directly linked to access to health services. Control of these NCD requires protocols that incorporate these results and promote action to rationalize drug use among specific populations that are easily identifiable in the system (older adults with heart disease and diabetes, for example), as well as action to encourage adherence among older adults to prescriptions which often contain several medicines with varied administration schedules. For such, it is necessary to monitor chronic treatments in primary care with action geared towards the prescription and dispensing of medicines, improving patient understanding and increasing successful use^2^.

Medical practice should be influenced by knowledge of prevalent polymorbidity and polypharmacy in older adults, looking for ways to therapeutically manage chronic morbidity that avoid iatrogenic diseases and side effects and maximize control of NCD, avoiding unnecessary hospitalizations and trips to the emergency room and, above all, the gradual incapacitation for daily activities and consequent loss of independence and autonomy^23^.

Despite the limitations inherent to a cross-sectional study in terms of causality inferences and the expected biases (recall bias in relation to medicines), PNAUM produced new, consistent and nationally representative data on the prevalence of use of chronic-use medicines by older adults, linked to specific NCD. The results show an expressive drug use among older adults, with polypharmacy in a sixth of them, with regional differences, and differentiated importance of some diseases, such as diabetes and heart diseases, in the amount of medicines used. Although there are studies showing that the deleterious effects of polypharmacy would really start at 10 drugs a day, most studies define polypharmacy with half that quantity^25^ ^-^ ^27^.

A surprising finding is that only 10 medicines, all in the RENAME list, account for almost half of the reports of chronic use of medicines by older adults.

The use of chronic-use medicines by older adults is an important dimension to be considered in the care of older adults, and polypharmacy appears as an indicator of need for safer and more effective use of medicines, avoiding the risk of iatrogenic diseases, side effects and worsened physical functioning^5^ ^,^ ^26^ ^,^ ^27^. Older adults with specific diseases have risk factors for polypharmacy which are modifiable by action aimed at the rational use of medicines. With the current population aging and the successful drug access policy via SUS, the trend is for drug use by older adults to grow further, potentially increasing costs, and thus should figure as a priority in the planning agenda of SUS.

## Footnotes

a Instituto Brasileiro de Geografia e Estatística. Pesquisa nacional de saúde: 2013: percepção do estado de saúde, estilos de vida e doenças crônicas: Brasil, grandes regiões e unidades da federação. Rio de Janeiro (RJ): IBGE; 2014.

b Ministério da Saúde, Secretaria de Ciência e Tecnologia e Insumos Estratégicos, Departamento de Assistência Farmacêutica. Relação Nacional de Medicamentos Essenciais: Rename; 2013. 8a ed. Brasília (DF): Ministério da Saúde; 2013 [cited 2016 Mar 23]. Available from: http://portalsaude.saude.gov.br/index.php/o-ministerio/ principal/leia-mais-o-ministerio/346-sctie-raiz/daf-raiz/farmacia-popular/l1-farmacia -popular/9681-lista-de-medicamentos-e-correlatos

c Reports on specific chronic-use medicines for each disease revealed two types of inconsistencies: the reported medicine could never be used for that disease and was considered a mistake due to the lack of knowledge of the older adult or the interviewer about the adequacy of the medication, which was probably indicated for a different morbidity; the drug was not specific for that disease, but in practice is often prescribed for patients with that disease (e.g., simvastatin for diabetics), and in these cases was considered non-specific. This created a classification, by medical and pharmaceutical consensus among researchers, of three types of medicines: specific, non-specific and innapropriate.
